# Quality-of-life and self-perceived inequity in people with severe mental illness: the SOFIA pilot study

**DOI:** 10.1080/02813432.2026.2697923

**Published:** 2026-08-11

**Authors:** Maarten Bjoern-Rozing, Volkert Siersma, Kristine Bissenbakker, Maria Haahr Nielsen, Alexandra Brandt Ryborg Jønsson, Anne Møller, John Brandt Brodersen

**Affiliations:** a Centre of General Practice, Department of Public Health, University of Copenhagen, Copenhagen, Denmark; b Research Unit for General Practice, Department of Community Medicine, Faculty of Health Sciences, UiT The Arctic University of Norway, Tromsø, Norway; c Department of People and Technology, Roskilde University, Roskilde, Denmark; d Research Unit for General Practice, Slagelse/Køge, Denmark

**Keywords:** Quality of life, mental health, psychiatry, patient-reported outcome measures, psychometrics

## Abstract

**Background:**

Living with severe mental illness (SMI) adversely affects quality of life (QoL). The SOFIA program, a coordinated general practice care initiative in Denmark, aimed to improve QoL and reduce mortality through extended general practitioner (GP) consultations, training for GPs and staff, and support in connecting patients with relevant health and social services.

**Objective:**

To evaluate the impact of the SOFIA intervention on needs-based QoL, self-perceived inequity, and health-related QoL in people with SMI.

**Design and Setting:**

Cluster-randomised, non-blinded controlled pilot trial conducted in nine Danish general practices between November 2020 and March 2021. Practices were allocated to a coordinated care program (CCP; *n* = 27), CCP plus a needs-based QoL tool (CCP+; *n* = 37), or usual care (control; *n* = 23).

**Subjects:**

Adults with psychotic disorders, bipolar disorder, or severe depressive disorder.

**Main Outcome Measures:**

Outcomes were assessed using the Multi-Morbidity Questionnaire (MMQ) and the EQ-5D-5L.

**Results:**

No statistically significant differences were observed between intervention and control groups in changes in needs-based QoL. Compared with the control group, the CCP+ group showed statistically significant improvements in two self-perceived inequity domains: *not being seen and heard and powerlessness.* No significant differences were found for the remaining inequity domains or health-related QoL.

**Conclusion:**

The findings suggest that comprehensive, coordinated GP consultations may help reduce feelings of inequity among people with SMI. Larger pragmatic randomised controlled trials with adequate power and longer follow-up are needed to determine the intervention’s effectiveness on patient-reported outcomes and long-term health.

**Trial registration**

The pilot trial protocol registration was dated 05/11/2020, and the registration number is NCT04618250.

## Background

Living with a severe mental illness (SMI), defined here as the conditions of severe depression, schizophrenia, or bipolar disorder, is known to impact an individual’s quality of life (QoL) negatively [1]. The reasons for impaired QoL are multifactorial and arise from the interplay between various factors associated with living with an SMI. These include an increased risk of chronic physical illnesses [2,3], psychiatric comorbidities [4], adverse medication effects, impaired access to health care [5] and social isolation [6]. This situation is further exacerbated by social factors often associated with mental illness, such as unemployment, low income, limited education, and not living with a partner [7]. Additionally, individuals with SMI often face stigma in the public sphere, socially, and when interacting with the healthcare system [8–10]. Self-reported outcomes suggest this stigma directly affects their QoL [11], adding to the burden of illness. Stigma is recognised as a key driver of health inequities and is regarded as a social determinant of health [12] that should be considered alongside other major social determinants. Epidemiological data show that people with SMI have a shorter life expectancy compared to the general population [13], with most deaths resulting from chronic physical conditions such as cardiovascular diseases, respiratory diseases, or cancer [14].

In Denmark, general practitioners (GPs) play a pivotal role in addressing the challenges faced by people with SMI as they serve as both gatekeepers to the secondary healthcare system and as coordinators of patient treatment and care. The Danish healthcare system is tax-funded and provides universal access to care, with GPs serving as the primary point of contact and gatekeepers to specialized services. Almost all citizens are listed with a GP, who is responsible for ongoing, comprehensive, and coordinated care. GPs are remunerated through a combination of capitation and fee-for-service, which may influence consultation patterns, including the duration and type of consultations provided. Patterns of healthcare utilization suggest that people with SMI rely more heavily on acute and emergency services [*15*], while engagement with planned primary care, including general practitioner (GP) consultations, is often suboptimal or inconsistent [*16*]. At the same time, inequities in access to and continuity of primary care persist, with fragmentation between services contributing to gaps in treatment and follow-up. These challenges are associated with unmet physical health needs and poorer overall outcomes, including reduced quality of life [*1*]. Strengthening the role of primary care and improving continuity of GP involvement may therefore represent an important pathway to enhancing quality of life and reducing health inequalities in this population.

The SOFIA program (short for **S**evere Mental Illness and Physical Health **I**n General Practice, or *Sammen Om Fysisk og psykisk helbred I Almen praksis* in Danish), a coordinated general practice care initiative developed specifically for general practice, aims to reduce mortality and enhance quality of life for people with SMI in Denmark. Created through a two-year participatory co-design process [17], the program seeks to strengthen the relationship between patients with SMI and their GP by aligning health care services with the individual preferences and needs of each patient. The core component of the programme was an extended, structured consultation between patients with SMI and their GP. Its feasibility was initially tested in a small-scale one-arm study in general practice [18] which demonstrated potential but also revealed certain barriers. As a result, the SOFIA programme was further refined and evaluated in the SOFIA Pilot Study, a cluster-randomised three-arm trial in general practice designed to inform the development of a future, large-scale randomised trial [19,20]. The two intervention arms received the extended consultation, while the one control arm did not. The SOFIA Pilot Study simultaneously collected data on health-related QoL, needs-based QoL, and self-perceived inequity with the latter two based on questionnaires specifically developed and validated for the SOFIA program. These assessments were conducted both before and after an extended consultation, or, in the case of the control group, three months apart [21]. In one of the two intervention arms, the pre-consultation assessment of needs-based QoL was used as a conversation tool during the extended consultations. In this article, we aimed to evaluate the impact of the extended structured consultation on needs-based QoL, self-perceived inequity and health-related QoL in people with SMI. The research question was twofold.

1. Are the changes in needs-based as well as health-related quality of life and self-perceived inequity observed in the SOFIA coordinated care program (CCP), with and without a needs-based QoL tool (CCP+), in general practice for individuals with severe mental illness, sufficiently sensitive for evaluation purposes?
2. Are there indications that adding a needs-based QoL tool (CCP+) may influence PROM outcomes compared with CCP alone?

We hypothesised that implementation of the SOFIA intervention with and without a needs-based QoL tool (CCP/CCP+) in general practice would be feasible. Moreover, we postulated that participants receiving CCP/CCP+ might show trends toward improvements in needs-based quality of life, self-perceived inequity compared with the control group. Finally, we expected that using a needs-based QoL tool (CCP+) during consultations would influence PROM.

## Material and methods

The present study is part of the SOFIA pilot trial, a cluster-randomised controlled study conducted in Danish general practice to assess the feasibility and potential impact of a coordinated care intervention for individuals with severe mental illness (SMI). The intervention includes extended GP consultations, training of general practitioners and practice staff, and support for coordinated, patient-centred care. The primary aim of the pilot trial is to evaluate the feasibility of the intervention and trial procedures, while exploratory analyses examine potential changes in patient-reported outcomes, including needs-based quality of life, self-perceived inequity, and health-related quality of life. These exploratory outcomes are intended to inform the design of a future definitive trial. The randomised SOFIA pilot study’s protocol and feasibility have been described in detail in two published articles [17,19]. Therefore, this section solely focuses on the methodology relevant to its specific aims of this article.

### Study design and setting

The study design was a cluster-randomised, non-blinded, parallel-group, three-armed trial. Patients with SMI connected to a specific general practice were allocated to one of two intervention groups (Coordinated Care Program (CPP) or CPP plus needs-based QoL as conversation tool (CCP+)) or a control group. The participating general practices located in the Eastern part of Denmark (the Capital Region of Denmark and the Region of Zealand) were randomly assigned to one of the three interventions using a computer algorithm on a 1:1:1 basis. Due to the nature of the study, it was impossible to blind the participants or other members of the research team [20].

### Stratification of eligible patients

- Eligible patients from the participating general practices were aged 18 and over on the day of inclusion of the general practice
*And*
- Had a psychotic disorder
  [Registered at general practice with International Classification of Primary Care version 2 (ICPC-2) psychiatric diagnosis p72 (psychotic disorders) which roughly corresponds to International Classification of Diseases 10 (ICD-10) codes F20-F29.9]
*Or*
- Had a bipolar disorder
  [Registered at general practice with ICPC-2 diagnosis of p73, bipolar mood disorders, roughly corresponding to ICD-10 codes F30-F31, and F34.0; *Or* have a prescription of lithium (Anatomical Therapeutic Chemical classification (ATC) N05AN); *Or* are registered at general practice with ICPC-2 diagnosis p76, unipolar depressive disorders, roughly corresponding to ICD-10 codes F32.2 - F32.3, and/or F33.2- F33.3, and F53.0; *And* have a prescription of Lamotrigine (N03AX09) and/or Carbamazepine (N03AF01) and/or Valproic acid (N03AG01)]
*Or*
- Had a severe depressive disorder.

[Registered at general practice with ICPC-2 diagnosis p76, unipolar depressive disorders roughly corresponding to ICD-10 codes F32.2 - F32.3, and/or F33.2- F33.3, and F53.0; *And* have a prescription of tricyclic antidepressants (ATC: N06AA) and/or Selective Norepinephrine Receptor Inhibitors (SNRI) venlafaxine (N06AX16) and duloxetine (N06AX21) and/or Monoamine Oxidase A Inhibitor (MAOI) (N06AG) and/or non-selective MAOI (N06AF).] As information on depression severity is not available within the general practice diagnostic coding system. which does not distinguish between mild, moderate, and severe depression, we used treatment patterns as a proxy for severity. Specifically, treatment with SNRIs or TCAs was considered indicative of more severe or treatment-resistant depression compared with SSRI treatment. This assumption reflects routine clinical practice, where SNRIs or TCAs are typically prescribed following insufficient response to first-line SSRI therapy or in the context of more complex clinical presentations, and thus may signal greater symptom burden or reduced treatment response.

Patients were excluded from participation if they were (A) subjected to any type of legal measure as stipulated in the Danish Mental Health Care Act (Danish: *Psykiatriloven*), e.g. forced detention or medication; (B) registered with a dementia diagnosis ICPC-2 p70, roughly corresponding to ICD-10 F0.01-0.03 or registered with organic psycho-syndrome or other neurological diseases (ICPC-2 P71, N73, N99); (C) receiving end-of-life care; (D) non-Danish speakers; (E) psychiatric diagnosis appeared to be incorrect or outdated; (F) assumed by the patient’s GP to have an overall functional level that is too low for meaningful participation in the study.

The patients were classified based on their most severe psychiatric diagnosis, ranking psychotic disorder as highest and depressive disorder as lowest. These diagnoses were mutually exclusive.

A computer algorithm selected a random sample of 45 patients who met the inclusion criteria in each general practice. Each sample contained three groups of 15 patients who fulfilled the diagnostic criteria regarding each of the three disorders/disease groups, that is a psychotic disorder, bipolar disorder or severe depressive disorder.

The GPs were instructed to verify the diagnosis for each of the sampled patients, assess patient eligibility, and recruit at least six patients: two patients from each of the three diagnostic subgroups. A reimbursement limit of 15 patients per practice was used. The GPs or practice staff contacted potentially eligible patients and invited them to an introduction visit, where they received information about the study and provided written informed consent if they wished to participate. Furthermore, the patients were invited to complete the MultiMorbidity Questionnaire (MMQ) and EQ-5D (EuroQoL) for the first time during the enrolment period from November 2020 until March 2021).

### Content of the SOFIA programme in the three arms of the study

The participating practices were divided into three groups: CCP, CCP+ and the control group. CCP received the coordinated care program containing the following three components: (1) Education of the GPs and their staff (*via* a mandatory one-day introductory course; (2) A specialised handbook provided for the GPs which contained contact information of relevant social and healthcare actors, referral options and helplines (in case of substance abuse, or risk of suicide or self-harm), developed specifically to the municipal and regional affiliation; (3) Reimbursement for an extended consultation for the patients with their GP following the SOFIA scheme, a structured consultation model. The control group received usual care, consisting of standard GP consultations without any structured extension, additional tools, or specific training. These consultations were conducted according to routine clinical practice and did not include the use of MMQ or a predefined consultation framework.

The intervention consisted of a single extended consultation, with the possibility of follow-up consultations. In brief, the extended consultation was held by the general practitioner and followed the SOFIA scheme, which comprises a further development of the existing consultation practice and communication skills as taught during Danish general practitioners’ specialty training. The consultation primarily focused on somatic health problems but could include discussing non-medical issues if most pressing for the patient. The consultations lasted up to 45 min, which included time for preparation, prescribing paraclinical testing, and referrals. The extended consultation was held by a GP, however paraclinical tests and other indicated point-of-care tests could be performed by practice staff either before or after the consultation.

Moreover, the CCP and CCP+ groups received an extended consultation based on a structured framework. This framework guided the consultation to cover key domains, including somatic health, mental health, and patient priorities. The MMQ was used as a supportive tool within the consultation to facilitate dialogue, helping to identify areas of concern and guide prioritisation and shared decision-making, rather than functioning as a strict decision algorithm. Participating GPs received training before the intervention, which included an introduction to the model’s rationale, instruction in the structured consultation format, and guidance on interpreting and using MMQ results in clinical practice. We have clarified the content and format of this training in the revised manuscript. Practices in CCP+ arm received the same coordinated care programme as well as an additional fourth component; the GPs were provided the sum scores from their patients’ assessment of needs-based QoL (MMQ1) before the extended consultation. The data was presented in a bar chart visualising the different dimensions of needs-based QoL along with a description of each scale (Appendix 1). The GPs in the CCP+ group were trained in an online seminar to use the bar chart as a guide for identifying themes to discuss during the patient-centred consultation process. The practices in the control group provided standard care for their patients with SMI, which in Denmark includes free access to primary health care.

### Outcomes

Two Patient-Reported Outcome Measures (PROMs) were used in the pilot study, MMQ [22] and EQ-5D^23^. The MMQ is a PROM in two parts developed and psychometrically validated to measure needs-based QoL (MMQ1) and self-perceived inequity (MMQ2) specifically in patients with multimorbidity, including psychiatric disorders. MMQ1, which reflects how the patients’ ability to meet their needs regarding QoL is affected by their conditions [21–23], is measured across six dimensions: Physical ability, Worries, Limitations in everyday life, Social life, Self-image and Personal finances. A higher sum score across these dimensions indicates a lower level of needs-based QoL [23].

MMQ2 measures self-perceived inequity [11] specifically how the patients perceive they are treated by their GP or other health care professionals, local authorities, family and friends, due to their illnesses [20,23]. MMQ2 covers four dimensions of experiences: (1) “feeling stigmatised”; 2) “not being seen and heard”; (3) “insufficient understanding of their disease burden”; (4) “feeling powerless”. A higher sum score indicates a higher level of self-perceived inequity [21,23]. Tables 3 and 4 provide an overview of the MMQ scales and scale scores.

The EQ-5D is a generic PROM developed by the EuroQoL Group to assess health-related QoL (HRQoL) in clinical and economic evaluations [24]. It comprises five items addressing mobility, self-care, usual activities, pain/discomfort and anxiety/depression, along with a visual analogue scale (EQ-VAS). Each item offers five response options: no problems, slight problems, moderate problems, severe problems and extreme problems, which are scored from 1 to 5, with higher scores indicating lower HRQoL. Additionally, EQ-VAS allows patients to rate their health on a scale from “the worst health you can imagine” to “the best health you can imagine” with scores ranging from 0 to 100. In this scale, higher scores indicate higher HRQoL [24].

All included patients were asked to complete the two PROMs at home twice: at baseline in March 2021 (before randomisation) and at the end of the trial in October 2021. In addition, the patients in CCP and CCP+ were invited to complete MMQ before the extended consultation.

### Data collection and statistical analysis

The SOFIA pilot study was designed primarily to assess the feasibility and acceptability of the intervention and study procedures, including recruitment, retention, and implementation in a real-world setting. The study was not powered to detect statistically significant differences in clinical outcomes. For each of the two PROMs it was recorded how many patients initiated the assessments (i.e. responded to at least one item), and how many patients completed them (i.e. responded to all items) at different time points: baseline, the end of the study, and for the CCP and CCP+ group immediately before the consultation. For each element of the PROMs – the various dimensions of MMQ and the five EQ-5D items, along with the EQ-VAS – we calculated the mean and the corresponding standard deviation (SD) for each time point.

Due to the relatively small sample size and the cluster-based design, there is a potential risk of indirect identification of participants, particularly when combining demographic and clinical characteristics. However, all data were handled and reported in accordance with data protection regulations, and results are presented in aggregated form to minimize this risk.

As this was a pilot study, analyses were primarily descriptive and exploratory, focusing on feasibility outcomes and exploratory estimates rather than formal hypothesis testing. Participants were analysed according to their allocated study group, and analyses accounted for clustering at the practice level. These means were analysed in linear mixed models relative to the three randomisation groups. We used a random patient effect to account for the non-independence of observations arising from the same patients at different time points, while a random practice effect was used to address the correlation of data from the same practice. All analyses were adjusted for patients’ sex and age. Reported effect estimates were the differences in change in responses from baseline to the end of the study between the intervention groups and the control group. The linear mixed model is implicitly robust to possible bias from differential attrition and we did not take additional measures to adjust for such bias. The PROM data were collected and managed using REDCap^®^ (Research Electronic Data Capture) [25]. REDCap^®^ is a secure, web-based software platform designed to support data capture for research studies. The data was analysed in SAS^®^ version 9.4.

## Results

### Recruitment and retention

Of the 138 practices contacted by e-mail and telephone by the research team, 12 practices accepted the invitation and were included in the pilot study [19]. In total, 92 patients gave consent to participate. Three practices dropped out during the study, one from each group (1 CCP, 1 CCP+ and 1 control group). The reasons for their withdrawal were doctors’ illness, inability to participate in the mandatory training course, and organisational changes within the practice. This resulted in six intervention practices and three control practices with a total of 87 included patients [19] (Table 1).

**Table 1.** Baseline characteristics of the survey respondents.

	CCP	CCP+	Control
	*(n = 27)*	*(n = 37)*	*(n = 23)*
Age, *n* (%)			
20–40 years	8 (29.6%)	12 (32.4%)	7 (30.4%)
40–60 years	7 (25.9%)	19 (51.4%)	8 (34.8%)
60–80 years	12 (44.4%)	6 (16.2%)	8 (34.8%)
Age (years), mean (SD)	51.9 (16.9)	46.1 (14.4)	48.9 (15.7)
Sex, n (%)			
Woman	17 (63.0%)	23 (62.2%)	18 (78.3%)
Man	10 (37.0%)	14 (37.8%)	5 (21.7%)
Other	0 (0.0%)	0 (0.0%)	0 (0.0%)
Inclusion diagnosis, *n* (%)			
Severe depression	13 (48.2%)	12 (32.4%)	10 (43.5%)
Bipolar disorder	7 (25.9%)	14 (37.8%)	8 (34.8%)
Schizophrenia	7 (25.9%)	11 (29.7%)	5 (21.7%)
Region, *n* (%)			
Capital	16 (59.3%)	20 (54.1%)	13 (56.5%)
Zealand	11 (40.7%)	17 (45.9%)	10 (43.5%)

### Characteristics of respondents

There were 27 respondents in the CCP, 37 in CCP+ and 23 in the control group. In the total sample, the mean age of the respondents ranged from 46.1 to 51.9 years, most of them were female, lived in the Capital Region of Denmark and the most prevalent psychiatric condition was severe depression (Table 1).

### Response rates

Patients in the CCP group had the highest baseline response rate at 92.6% (Table 2). Response rates declined in all three groups by the end of the study to 55%–70%, the lowest at 54.1% for completion of MMQ2 by CCP+. The response rate at the study end was consistently lowest in the CCP+ group.

**Table 2.** Response rates of partially- and fully completed PROMs.

	Baseline (*n*,%)		Consultation (*n*,%)		End of study (*n*,%)	
	Initiated	Completed	Initiated	Completed	Initiated	Completed
MMQ1						
CCP	25 (92.6%)	24 (88.9%)	23 (85.2%)	22 (81.5%)	21 (77.8%)	21 (77.8%)
CCP+	31 (83.8%)	28 (75.7%)	25 (67.6%)	25 (67.6%)	21 (56.8%)	21 (56.8%)
Control	19 (82.6%)	13 (56.5%)			16 (69.6%)	15 (65.2%)
MMQ2 (GP)						
CCP	25 (92.6%)	25 (92.6%)	20 (74.1%)	19 (70.4%)	20 (74.1%)	20 (74.1%)
CCP+	30 (81.1%)	28 (75.7%)	24 (64.9%)	23 (62.2%)	21 (56.8%)	20 (54.1%)
Control	18 (78.3%)	16 (69.6%)			15 (65.2%)	14 (60.9%)
EQ-5D						
CCP	25 (92.6%)	25 (92.6%)			21 (77.8%)	20 (74.1%)
CCP+	31 (83.8%)	31 (83.8%)			21 (56.8%)	21 (56.8%)
Control	19 (82.6%)	18 (78.3%)			16 (69.6%)	15 (65.2%)

### Effect of the intervention

#### Effect of the intervention on needs-based QoL measured with MMQ1

Throughout the study period, there were no differences in the change of the score across any of the MMQ1 scales between the intervention groups and the control group (Table 3, Supplementary Figure).

#### Effect of the interventions on self-perceived inequity measured with MMQ2

In the CCP+ group, differences were observed during the study period in two scales related to contacts with the GP: “Experience of not being ‘seen and heard’” (*p* = 0.025) and “Experience of powerlessness” (*p* = 0.031). For the other MMQ2 scales, no statistically significant differences were found throughout the study period among respondents in the two intervention groups regarding their encounters with the GP (Supplementary Figure and Table 4). Additionally, there were no statistically significant differences over the study period in interactions with staff at the GP, other healthcare professionals, local authorities, friends, family, or others (Appendix 2).

**Table 3.** Needs-based QoL (MMQ1) in the intervention and control groups.

	Baseline		Consultation		End of study		Difference in change over study period	
	*n*	Mean (SD)	*n*	Mean (SD)	*n*	Mean (SD)	Mean (95% CI)	*p*-value
Limitations in physical ability (range: 0–18)								
CCP	24	8.00 (4.67)	22	7.09 (4.59)	21	7.29 (4.78)	1.76 (−0.88; 4.40)	0.1880
CCP+	30	7.93 (5.34)	25	9.08 (4.45)	21	7.43 (4.93)	0.98 (−1.65; 3.60)	0.4630
Control	17	7.53 (4.67)	–		16	5.44 (4.27)	(ref)	
Worries (range: 0–18)								
CCP	25	8.96 (4.33)	23	9.22 (3.19)	21	9.14 (4.65)	1.03 (−1.38; 3.43)	0.3983
CCP+	30	10.50 (5.42)	25	10.68 (4.75)	21	9.95 (4.97)	−0.56 (−2.96; 1.84)	0.6449
Control	19	8.68 (5.08)	–		16	7.81 (5.47)	(ref)	
Limitations in everyday life (range: 0–30)								
CCP	25	13.44 (7.99)	23	12.83 (6.98)	21	12.86 (7.52)	−0.01 (−3.45; 3.44)	0.9979
CCP+	31	13.23 (9.35)	25	15.04 (8.21)	21	12.52 (8.30)	−0.73 (−4.17; 2.72)	0.6769
Control	15	11.60 (7.60)	–		16	9.88 (8.04)	(ref)	
Limitations to social life (range: 0–18**)**								
CCP	24	6.42 (4.24)	23	5.91 (4.29)	21	6.57 (3.79)	1.36 (−0.89; 3.60)	0.2326
CCP+	31	7.39 (5.40)	25	8.08 (4.74)	21	6.52 (4.25)	−0.04 (−2.26; 2.19)	0.9750
Control	18	6.11 (4.66)	–		15	4.80 (5.54)	(ref)	
Negatively affected self-image (range: 0–18)								
CCP	25	8.64 (5.12)	23	7.17 (4.37)	21	7.71 (4.30)	0.54 (−1.65; 2.72)	0.6278
CCP+	30	9.77 (5.61)	25	10.60 (5.02)	21	9.76 (5.19)	1.35 (−0.83; 3.53)	0.2222
Control	18	8.17 (5.50)	–		15	6.40 (4.85)	(ref)	
Worries about personal economy (range: 0–9)								
CCP	25	3.12 (2.83)	23	2.48 (2.54)	21	2.48 (2.60)	0.18 (−1.00; 1.36)	0.7607
CCP+	30	4.07 (3.33)	25	4.52 (3.55)	21	4.57 (3.60)	1.12 (−0.06; 2.29)	0.0632
Control	18	4.22 (3.42)	–		15	3.40 (3.32)	(ref)	

Note: The table provides the Needs-based QoL (MMQ1) mean scale scores of the intervention groups and the control group (at baseline, at the consultation, and by the end of the study) and a comparison of the score differences over the study period**. A high score indicates a low level of needs-based QoL.**

**Table 4.** Self-perceived inequity in intervention and control groups.

	Baseline		Consultation		End of study		Difference in change over study period	
	*n*	Mean (SD)	*n*	Mean (SD)	*n*	Mean (SD)	Mean (95% CI)	*p*-value
Experience of not being “seen and heard” when in contact with my GP (range: 0–12)								
CCP	25	0.64 (1.41)	20	0.65 (0.93)	20	0.60 (1.60)	−0.90 (−1.85; 0.04)	0.0614
CCP+	30	0.63 (1.47)	23	0.48 (1.35)	21	0.38 (1.20)	−1.07 (−2.01; −0.14)	***0.0248***
Control	17	0.53 (1.33)	–		15	1.53 (2.42)	(ref)	
Experience of being stigmatized when in contact with my GP (range: 0–15)								
CCP	25	0.24 (0.52)	20	0.15 (0.49)	20	0.20 (0.52)	0.11 (−0.59; 0.81)	0.7566
CCP+	30	0.67 (1.58)	24	0.50 (1.25)	21	0.29 (0.90)	−0.14 (−0.82; 0.55)	0.6914
Control	17	0.65 (1.22)	–		15	0.53 (1.06)	(ref)	
Experience of insufficient understanding of the burden of disease when in contact with my GP (range: 0–9)								
CCP	25	0.56 (1.00)	19	0.79 (1.13)	20	0.70 (1.53)	−0.04 (−0.98; 0.90)	0.9307
CCP+	29	0.62 (1.21)	24	0.58 (1.47)	21	0.90 (2.12)	0.26 (−0.66; 1.19)	0.5729
Control	18	0.50 (0.92)	–		14	0.71 (1.54)	(ref)	
Experience of powerlessness when in contact with my GP (range: 0–15)								
CCP	25	0.56 (1.12)	20	0.50 (0.83)	20	0.50 (1.19)	−0.94 (−1.96; 0.09)	0.0735
CCP+	29	0.93 (1.79)	24	0.58 (1.47)	20	0.70 (1.53)	−1.13 (−2.16; −0.10)	***0.0314***
Control	17	0.71 (1.96)	–		15	1.73 (3.28)	(ref)	

Note: The table provides the Self-perceived inequity, when in contact with my GP (MMQ2 (GP)) mean scale scores of the intervention groups and the control group (at baseline, at the consultation and by the end of the study) and a comparison of the score differences over the study period. **A high score indicates a high level of self-perceived inequity**.

#### Effect of the intervention on HRQoL measured with EQ-5D

There was no statistically significant change in health status in the intervention groups over the study period (Table 5).

**Table 5.** Health-related quality of life (EQ-5D) for the intervention and control groups.

	Baseline		Consultation		End of study		Difference in change over study period	
	*n*	Mean (SD)	*n*	Mean (SD)	*n*	Mean (SD)	Mean (95% CI)	*p*-value
Mobility (range: 1–5)								
CCP	25	1.72 (0.84)	–		21	2.05 (1.16)	0.24 (−0.36; 0.85)	0.4197
CCP+	31	1.74 (1.03)	–		21	1.86 (0.96)	−0.03 (−0.64; 0.57)	0.9125
Control	18	1.67 (0.69)	–		16	1.75 (1.00)	(ref)	
Self-care (range: 1–5)								
CCP	25	1.24 (0.52)	–		21	1.38 (0.67)	−0.00 (−0.47; 0.47)	0.9947
CCP+	31	1.39 (0.62)	–		21	1.57 (0.68)	0.03 (−0.43; 0.49)	0.9024
Control	18	1.28 (0.57)	–		16	1.44 (0.63)	(ref)	
Usual activities (range: 1–5)								
CCP	25	2.48 (1.00)	–		21	2.33 (1.06)	−0.41 (−1.10; 0.28)	0.2394
CCP+	31	2.35 (1.17)	–		21	2.81 (1.29)	0.12 (−0.57; 0.81)	0.7234
Control	18	2.00 (0.97)	–		16	2.19 (1.17)	(ref)	
Pain/discomfort (range: 1–5)								
CCP	25	2.52 (1.05)	–		21	2.52 (1.17)	0.26 (−0.37; 0.89)	0.4071
CCP+	31	2.52 (1.12)	–		21	2.38 (0.97)	0.12 (−0.51; 0.74)	0.7141
Control	18	2.39 (0.84)	–		16	2.13 (0.81)	(ref)	
Anxiety/depression (range: 1–5)								
CCP	25	2.56 (0.77)	–		21	2.24 (1.14)	−0.36 (−1.05; 0.33)	0.3001
CCP+	31	2.48 (1.09)	–		21	2.57 (1.29)	0.01 (−0.67; 0.70)	0.9661
Control	19	2.32 (1.11)	–		15	2.47 (0.92)	(ref)	
VAS (range: 0–100)								
CCP	25	53.36 (18.75)	–		20	59.85 (19.31)	3.48 (−11.75; 18.71)	0.6485
CCP+	31	52.84 (24.07)	–		21	53.52 (26.82)	−0.62 (−15.62; 14.37)	0.9338
Control	19	61.58 (21.66)	–		16	64.06 (19.02)	(ref)	

Note: The table provides the EQ-5D HRQoL mean scores for the intervention groups and the control group (at baseline and by the end of the study) and a comparison of the score differences within the groups over the study period. For the five EQ-5D items, a high value indicates a bad health state, in the EQ-5D VAS, a low score indicates a bad health state.

## Discussion

There were no statistically significant differences in the change in needs-based quality of life between the intervention and control groups. These findings suggest that using the MMQ1 as a conversation tool during consultations does not influence PROM outcomes for MMQ1 in the pilot study. However, we found an improvement in patients’ self-perceived inequity (MMQ2) in the domains of “feeling less powerless” and “being seen and heard” in the CCP+ group. A similar, though not statistically significant, trend was noted in the CCP group. This suggests that using MMQ1 as a communication prompt by the GP during extended consultations may help reduce perceived inequity among patients with SMI. However, it is important to note that this reduction is relative to the control group, which experienced a worsening of self-perceived inequity during the study. No statistically significant effects were found in the remaining PROM outcomes related to health-related quality of life (EQ-5D).

### Comparison with existing literature

The MMQ has not been used in any previous randomized intervention studies. We found in a systematic review that six PROMs were developed for patients with multimorbidity; however, they all possessed inadequacy in their measurement properties [26]. Therefore, we developed the MMQ. In previous trials the measurement of quality of life in people with multimorbidity has typically relied on generic utility measures such as EQ-5D-5L and SF36. Although EQ-5D-5L has well-established validity in many contexts, it was not designed for people with multimorbidity and has recognised psychometric limitations, including inadequate content validity. The reliance on generic QoL measures in multimorbidity research may be a contributory factor to the negative results of many multimorbidity intervention trials [27]. It has been shown that MMQ has a very high content validity [21] and adequate psychometric properties [23]. The MMQ has also shown its clinical relevance in a feasibility study [19]. Moreover, the UK English version of the MMQ1 has been compared with its ability to measure variations in quality of life with the EQ-5D-5L. We found that MMQ1 was superior to EQ-5D-5L in its ability to detect age-related variations in QoL and to discriminate between different levels of self-rated QoL. Thus we conclude that MMQ1 has the potential to improve the measurement of QoL in people with multimorbidity [27].

The lack of measurable effects (apart from the two scales related to self-perceived inequity) may be due to limitations in the ability of the applied PROMs, specifically EQ-5D and MMQ1, to detect changes among respondents. Given the well-established evidence that patients with SMI experience a lower quality of life [2], we anticipated that respondents in this study would exhibit high PROM sum scores, as higher scores on both MMQ1 and EQ-5D indicate a lower QoL. The mean of the responses to EQ-5D is between levels one to three (out of five), leading us to question the measurement capability of the EQ-5D for this patient group. EQ-5D is a generic utility measure developed for clinical and economic assessment of health; yet, along with Short-Form 36 (SF-36), it is the most frequently used utility measure of HRQoL in patients with chronic conditions [28]. Its strength lies in its practical applicability and widespread use, facilitating comparability between studies. Yet concerns have been raised about whether it can adequately capture the complexity of living with SMI. Generic measures in healthcare often have an exclusive focus on physical limitations and are not developed with patient input, leading to limited content validity in PROMs [29,30] and raising concerns about the relevance and comprehensiveness of what is being assessed [30]. Moreover, the psychometric properties of generic PROMs are questionable due to the potential for differential item functioning (DIF), where items may perform differently among respondent groups, e.g. the three randomization groups [31,32], rendering these groups incomparable on such PROMs. Given the diverse nature of patient groups with psychiatric conditions and somatic comorbidities, the risk of DIF is substantial, making the use of generic measures particularly problematic.

Since the MMQ was developed specifically for patients with multimorbidity, including psychiatric conditions, and has been evaluated for its psychometric properties, including DIF, its content and construct validity are considered suitable for measuring outcomes among the respondents in the SOFIA trial [8,21,22]. The MMQ’s limitation with regard to this pilot study lies in the limited discrimination ability in small sample sizes [23], a limitation which is not inherent to the MMQ itself. Discrimination expresses the number of individuals required in each study to find a clinically meaningful difference in the response categories. This measurement ability of MMQ1 has been assessed in a previous article and ranges from 34 to 10.478 depending on the scale and the response category (the discrimination ability tends to perform more poorly in the extreme ends of the response categories) [23]. In this pilot study with a sample size of 88, the scale’s ability to discriminate between responses is limited in the categories from “Very poor” to “Poor” and “Good” to “Very good.” While the discrimination ability of the MMQ2 has not yet been evaluated, we observed a significant difference over the study period in the scales measuring “Experience of not feeling seen and heard” and “Experience of powerlessness.” To our knowledge, the EQ-5D’s discriminative ability, particularly for assessing patients with SMI and somatic comorbidities, has not been tested.

### Strengths and limitations

The primary aim of the randomized SOFIA Pilot Study was not to measure changes in QoL for patients with SMI, but to assess the feasibility and fidelity of implementing and evaluating the designed intervention [17]. Considering these aims, it is encouraging that, despite the small sample size, there were indications that the extended SOFIA consultations may contribute to a lower degree of self-perceived inequity, a finding that merits further assessments in future studies. We acknowledge that the intervention combines several components, including extended consultation time, structured content, and MMQ-guided dialogue. As these elements were introduced together, the study does not allow teasing out the independent effect of extended consultation time from the structured approach. Interpretation of these effect estimates should be made with caution. Although the analyses suggest that the MMQ1 scales are capable of detecting clinically meaningful differences, particularly in the mid-range response categories, the precision and generalizability of these estimates are constrained by several factors. First, the relatively small sample size limits the stability of the estimates and increases the uncertainty around the calculated discriminating ability. Second, the short follow-up period restricts our ability to capture meaningful changes over time, which may be particularly relevant for outcomes expected to evolve gradually. Third, the study population consisted of relatively well-functioning patients, which likely reduced variability and introduced potential ceiling effects, as reflected in the substantially larger sample sizes required to detect differences at the higher end of the scale (e.g. between “good” and “very good”). Taken together, these factors suggest that while the estimates are useful for gauging plausible effect sizes and informing future study design, they should not be interpreted as definitive evidence of the instrument’s discriminative performance. While some outcomes suggest potential beneficial trends, these findings should be interpreted with caution given the small sample size and feasibility design.

No differences were detected in other PROM outcomes related to needs-based QoL and HRQoL. These negative findings could be due to several factors: (1) the small sample size (ranging from 9 to 31 respondents), which limited statistical power and increased the risk of a type 2 error; (2) the study’s pilot design and timeframe, which allowed only one extended consultation per patient- changes in need-based QoL may not be expected after just one consultation and only a 6-month follow-up, as this concerns more fundamental aspects; and (3) the recruitment process, which is skewed towards recruitment of patients with whom the GP already had a strong relationship, leaving little room for measurable improvement in self-perceived inequity (MMQ2) and thereby decreasing the statistical variance. This is a consequence of the potential selective inclusion of patients with SMI for the SOFIA pilot study [33]. The patients included in our pilot study showed a pronounced floor effect on all scales at the baseline measurement, suggesting that, from the outset, these individuals did not present with significant challenges. However, if applied in a context where patients face more severe and complex difficulties, the SOFIA consultation might have a larger impact on the MMQ scales and potentially also on HRQoL.

In Denmark, GP remuneration is based on a mixed model combining capitation and fee-for-service, where specific services, including consultations, are reimbursed according to nationally agreed tariffs. Changes in reimbursement structures may therefore influence GP behaviour, for example, by affecting the time allocated to individual patients or the prioritization of consultation types. This context is important when interpreting our findings, as financial incentives may have contributed to the observed patterns in GP consultations.

### Implications for research and practice

By implementing extended SOFIA consultations, where GPs use the MMQ as a conversation tool for people with SMI, we observed a positive impact on patients’ self-perceived inequity. These trends should be evaluated in a adequately powered RCT involving more GPs, additional patients with SMI and complex problems (higher scores on the MMQ scales), more frequent extended SOFIA consultations, and a longer follow-up period. The SOFIA Pilot Study aimed to assess the feasibility of the intervention to guide the RCT. However, as of January 1, 2022, reimbursement was introduced for extended consultations for all GPs. As a result, the planned RCT involving 200 practices in Denmark was cancelled. Although the agreement incorporated elements of the SOFIA program, it did not include the MMQ. Therefore, we aim to investigate the implementation of extended consultations and the use of the MMQ as a conversation tool in future research projects, including qualitative data collection from patients about their experiences with these extended consultations.

Recognizing the critical role GPs play in managing care and treatment for patients with SMI and somatic comorbidities, using the MMQ1 as a conversation tool helps structure consultations around QoL for this patient group. It aids GPs in identifying and prioritizing the issues most important to individual patients, fostering a patient-centred consultation and ensuring a more holistic approach to their often complex life situations. As a result, the MMQ project team is currently working on integrating the MMQ1 as a conversation tool into the Danish reimbursement system for GP consultations with patients with SMI. Additionally, assessing the use of MMQ1 as a conversation tool qualitatively with both GPs and patients with SMI would provide valuable insights.

## Conclusion

This small-scale randomized pilot study did not provide evidence that the SOFIA intervention had an impact on the needs-based QoL or HRQoL. However, patients in the intervention group using the MMQ as a conversation tool reported less self-perceived inequity in the two scales about being seen and heard and powerlessness. These findings suggest that the comprehensive approach in the SOFIA consultation may be essential to prevent individuals with SMI from experiencing feelings of inequity. *While some outcomes suggest potential beneficial trends, these findings should be interpreted with caution given the small sample size and feasibility design.*

## Supplementary Material

Supplemental Material
