# Supplementary material for: Quality-of-life and self-perceived inequity in people with severe mental illness: the SOFIA pilot study
Source: Scand J Prim Health Care. 2026 Aug 11;44(1):2697923. doi: 10.1080/02813432.2026.2697923 (PMC13463517; doi:10.1080/02813432.2026.2697923)
Supplement: Supplementary file 1 [file IPRI_A_2697923_SM7248.docx]

NB: Numerator in response rate calculations is based on patients enrolled by baseline
(n=64 in the intervention group and n=23 in the control group)
